# Supplementary material for: The causal relationship between the human gut microbiota and pyogenic arthritis: a Mendelian randomization study
Source: Front Cell Infect Microbiol. 2024 Nov 26;14:1452480. doi: 10.3389/fcimb.2024.1452480 (PMC11629706; doi:10.3389/fcimb.2024.1452480)
Supplement: Supplementary file 1 [file DataSheet1.zip › mendelian test/ebi-a-GCST90027575.csv_forest.pdf]

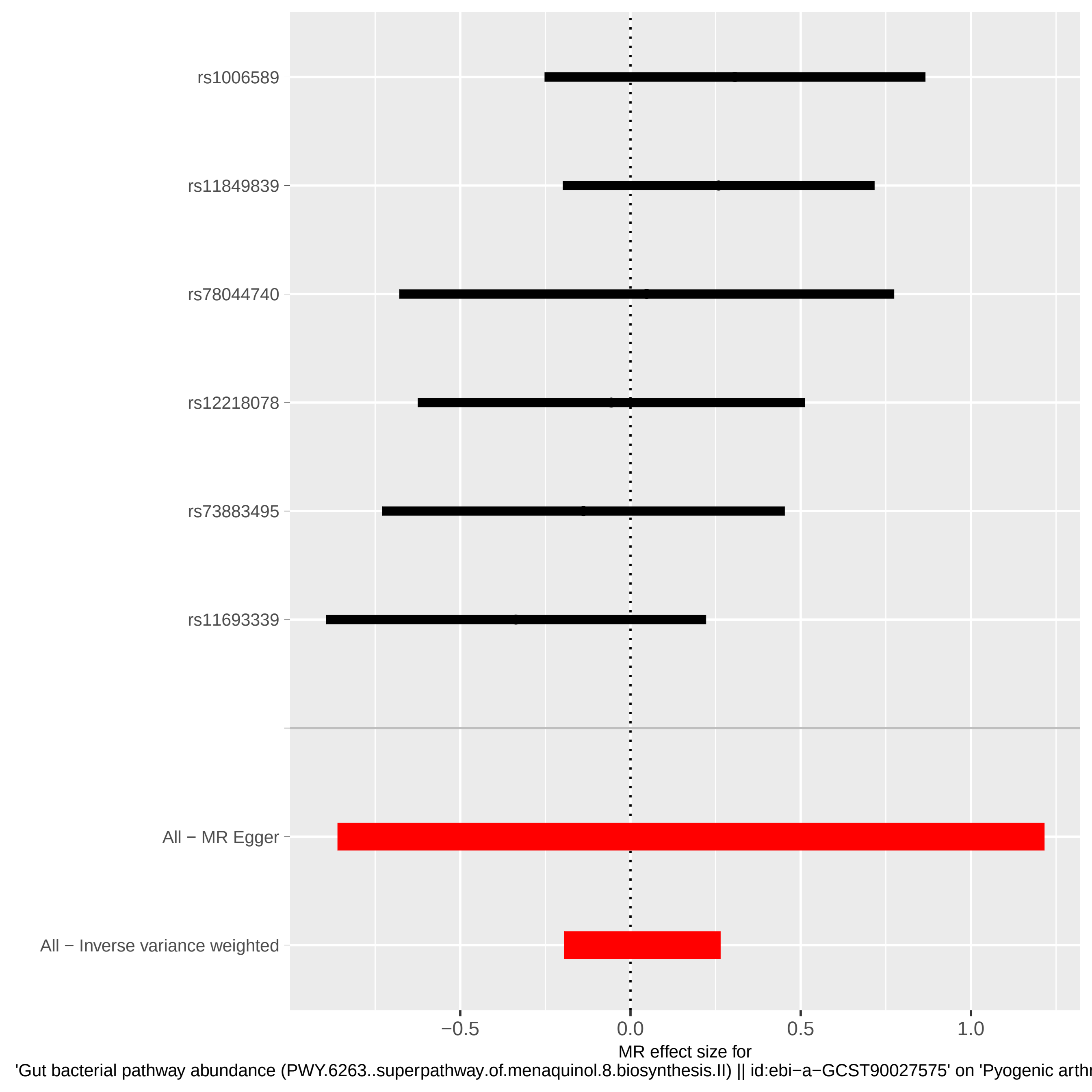
rs1006589
rs11849839
rs78044740
rs12218078
rs73883495
rs11693339
All − MR Egger
All − Inverse variance weighted
−0.5
0.0
0.5
1.0
MR effect size for
'Gut bacterial pathway abundance (PWY.6263..superpathway.of.menaquinol.8.biosynthesis.II) || id:ebi−a−GCST90027575' on 'Pyogenic arthr
